# Evaluation of Chemical Constituents of Litchi Pericarp Extracts and Its Antioxidant Activity in Mice

**DOI:** 10.3390/foods11233837

**Published:** 2022-11-28

**Authors:** Ziming Yang, Li Zhang, Yuan-Hang Wu, Dian-Peng Li, Wei Li

**Affiliations:** 1 Guangxi Key Laboratory of Plant Functional Phytochemicals and Sustainable Utilization, Guangxi Institute of Botany, Guangxi Zhuang Autonomous Region and Chinese Academy of Sciences, Guilin 541006, China; 2 School of Materials Science and Engineering, Sun Yat-sen University, Guangzhou 510006, China

**Keywords:** litchi pericarp, antioxidant effect, chemical constituents, polyphenols, mice

## Abstract

Litchi pericarp is the main byproduct of litchi processing and contains several polyphenols. However, the chemical constituents and the antioxidant effect in litchi pericarp extracts (LPE) have been rarely studied. The result of the quantitative analyses of the major monomers in LPE indicated that procyanidin A2, procyanidin B2, epicatechin, rutin, and catechin were the major polyphenol compounds of LPE. The LPE exhibited high radical scavenging activity, as indicated by the results of the 1,1-diphenyl-2-picrylhydrazyl (DPPH) and ascorbic acid, 2,2′-Azinobis-3-ethylbenzothiazoline-6-sulfonic acid (ABTS) tests. Moreover, administrating D-galactose in mice led to the reduced activity of antioxidant enzymes, aggravated lipid peroxidation, and induced protein oxidation. The results were improved in the aging mice after the LPE treatment was performed. The above results suggest that LPE has an excellent antioxidant effect. Accordingly, litchi pericarp can serve as a promising source of dietary antioxidants.

## 1. Introduction

Litchi (*Litchi chinensis* Sonn.) is a subtropical and tropical fruit of China, and it is now broadly cultivated in China and throughout the world [1,2]. The estimated total global production of litchi fruit was 3.50 million tons in 2022, and China has been the world’s largest producer thus far, with approximately 2.00 million tons. However, fresh fruits have a short harvest season and are perishable, and some fruits experience seasonal oversupply, thus causing a huge burden on the fresh fruit market. Litchi processing produces considerable pericarps and seeds. Litchi pericarp takes up about 15% of the whole fresh litchi weight [3]. In the past, the above by-products have been generally discarded as waste, which has seriously wasted resources and polluted the environment.

Flavonoids, polyphenols, proanthocyanidins, and anthocyanins have attracted increasing attention for their significant bioactivities (e.g., anti-inflammatory, antioxidant, antibacterial, and antitumor activities) [1]. Litchi fruit turns red when ripe. This color suggests that litchi pericarp may have a high content of polyphenols. Based on previous chemical investigations, litchi pericarp contains polyphenols, including (+)-catechin, epicatechin, rutin, quercetin, (-)-epigallocatechin, epigallocatechin gallate, 3,4-Dihydroxybenzoic acid, procyanidin A2, procyanidin B2, procyanidin A1, procyanidin B1, and cyanidin-3-*O*-glucoside chloride [4,5,6]. Accordingly, litchi pericarp is a promising research raw material and contains abundant active compounds. Litchi pericarp can serve as a preservative for fresh and frozen meat and meat products for its antioxidant properties [7]. The use of litchi pericarp in functional foods has aroused people’s interest [8]. Litchi pericarp serves as an excellent source of bioactive ingredients, and its utilization should be a technological priority in the management of by-product processing from environmental and economic perspectives.

Antioxidants are molecules used to prevent other compounds from being oxidized [5]. The antioxidant function possessed by individual compounds from litchi pericarp has been investigated in depth [3,5,9,10,11], whereas this is not the case for the whole litchi pericarp. Multiple components with complex interactions in litchi pericarp may result in significantly different outcomes as compared with individual constituents. According to this rationale, this study focused on the whole litchi pericarp extracts (LPE). Several reports have been published on the spectrum of antioxidant benefits of whole LPE [1,4,12], whereas the composition of the above extracts remains unclear, and the polyphenols content was very low. Nevertheless, little research has been undertaken regarding the in vivo antioxidant activity of whole LPE.

In this study, a novel method of polyphenols extraction for litchi pericarp was proposed. Furthermore, the contents of the major monomers in the LPE were obtained, and the antioxidant function of LPE in vitro and in vivo was examined.

## 2. Materials and Methods

### 2.1. Experimental Material

Sodium carbonate (Na_2_CO_3_), glacial acetic acid (CH_3_COOH), edible ethanol, absolute ethanol, potassium acetate (C_2_H_3_KO_2_), aluminum trichloride (AlCl_3_), potassium chloride (KCl), sodium acetate (CH_3_COONa), methanol, sulfuric acid (H_2_SO_4_), and potassium persulfate (K_2_S_2_O_8_) originated from Sinopharm Chemical Reagent Co. (Shanghai, China). Folin–Ciocalteu phenol reagent, 1,1-diphenyl-2-picrylhydrazyl (DPPH), ascorbic acid, 2,2’-Azinobis-3-ethylbenzothiazoline-6-sulfonic acid (ABTS), vitamin E (VE), and D-galactose were offered by Sigma-Aldrich Chemicals (St. Louis, MO, USA). PUSH BIO-TECHNOLOGY (Chengdu, China) provided: gallic acid, rutin, epicatechin, vanillin, formic acid, (+)-catechin, (-)-epigallocatechin, 3,4-dihydroxybenzoic acid, quercetin, epigallocatechin gallate, procyanidin A2, procyanidin B2, procyanidin A1, procyanidin B1, and luteolin. A malondialdehyde (MDA) assay kit, a superoxide dismutase (SOD) assay kit, a reduced glutathione (GSH) assay kit, and a protein carbonyl assay kit originated from Nanjing Jiancheng Bioengineering Institute (Nanjing, China). Pentobarbitol sodium was provided by HeFei BoMei Biotechnology Co. Ltd. (Hefei, China).

### 2.2. Sample Collection and Processing

Fresh litchi fruit was purchased from the Yanshan district farmers’ market, Guilin city, Guangxi Zhuang Autonomous Region, and identified by Weibing Xu (Guangxi Institute of Botany, Guangxi Zhuang Autonomous Region, and Chinese Academy of Sciences, Guilin). Subsequently, we deposited the litchi pericarp in the Guangxi Key Laboratory of Plant Functional Phytochemicals and Sustainable Utilization (voucher no: GXZW20190725). We maintained the litchi pericarp in bags at −80 °C for subsequent use.

### 2.3. Litchi Pericarp Extraction

We extracted polyphenols from litchi pericarp, as described below. In brief, the freeze drying of 500 g of litchi pericarp was performed in a freeze dryer. Subsequently, the lyophilized litchi pericarp was pulverized into a powder form using a grinder (GLZY-0.5B, Shanghai Pudong Freeze Drying Equipment Co., Ltd., Shanghai, China). We placed the litchi pericarp powder in a saturated aqueous Na_2_CO_3_ solution at pH = 10 (3 L) at 60 °C for 6 h. Next, the mixture passed through a 60-mesh filter cloth for filtering, and the residue continued in a saturated aqueous Na_2_CO_3_ solution at pH = 10 (1.5 L) at 60 °C for 6 h. Moreover, the mixture again passed through a 60-mesh filter cloth for filtering, and the filtrate was then combined, followed by 15 min of centrifugation at 4000 rpm/min in a desktop centrifuge (TGL-16R, Dark Horse Instrument and Equipment Co., Ltd., Zhuhai, China). Afterward, the collected supernatant was neutralized to pH 3 with CH_3_COOH, and the supernatant was loaded onto a food-grade HPD–100 macroporous resin column (Haoju Resin Technology Co., Ltd., Tianjin, China). Distilled water was employed for washing the column, and 30% edible ethanol was used for the elution. The collected ethanol eluent had a decompressing concentration in a rotary evaporator (Rikakikai Co., Ltd., Tokyo, Japan). The concentrated ethanol eluent was dried with a vacuum-freezing dryer, and LPE was obtained. Dried LPE was weighed to obtain the extraction rate. The LPE was employed for compound analysis and corresponding bioactivity assays.

### 2.4. Total Polyphenol Determination

The Folin–Ciocalteu phenol reagent method assisted in obtaining the total polyphenol content of LPE [13]. In brief, the gallic acid stock solution (concentration: 5 mg/mL) and the gallic acid working solution (standard concentration: 0–1000 μg/mL) were prepared in distilled water. The results are expressed as milligrams of gallic acid equivalents per gram of LPE (mg GAE/g LPE). A gallic acid standard solution of 0.15 mL was mixed with 1.5 mL of Folin–Ciocalteu phenol reagent. Shaking and 5 min of rest were followed by the addition of 1.5 mL of Na_2_CO_3_ aqueous solution (6% *w/v*). After the solution was stirred, the mixture underwent 10 min of incubation at 60 °C in a dark place. Subsequently, the solution was placed in an ice bath for being cooled to 0–4 °C, and a spectrophotometer was used to examine the absorbance at 725 nm (T6, General Instruments Co., LLC., Beijing, China). The LPE working solution (concentration: 1 mg/mL) was prepared in 50% ethanol, and the total polyphenol content of LPE was examined according to the above method. The procedure was repeated three times. The GAE standard curve and regression equation were established and the total polyphenol content of LPE was calculated according to the GAE standard curve.

### 2.5. Total Flavonoid Determination

The aluminum trichloride reagent method assisted in examining the total flavonoid content of LPE [14]. This method was implemented basically in the following processes: the rutin stock solution (concentration: 0.5 mg/mL) and the working solution (standard concentration: 0–50 μg/mL) were prepared in ethanol. The results are expressed as milligrams of rutin equivalents per gram of LPE (mg RE/g LPE). A rutin standard solution of 0.2 mL was mixed with 0.04 mL of C_2_H_3_KO_2_ solution (0.1 M), 0.6 mL of absolute ethanol, 1.8 mL of distilled water, and 0.04 mL of AlCl_3_ solution (10%, *w*/*v*). After stirring, the mixture underwent half an hour of incubation at ambient temperature in a dark place. After filtering treatment, a spectrophotometer was used to examine the absorbance at 415 nm. The LPE working solution (concentration: 0.1 mg/mL) was prepared in 50% ethanol, and the above method was also adopted for examining the total flavonoid content. The procedure was repeated three times. The rutin (RE) standard curve and regression equation were established and the total flavonoid content of LPE was calculated according to the RE standard curve.

### 2.6. Total Anthocyanin Determination

The pH–differential method assisted in examining the total anthocyanin content [15]. We mixed 0.5 mL of LPE (concentration: 10 μg/mL) with 4.5 mL of KCl buffer (pH 1.0) and examined the absorbance of the mixed solutions at 515 nm and 700 nm. Likewise, we mixed the 0.5 mL of LPE with 4.5 mL of CH_3_COONa buffer (pH 4.5) and examined the absorbance at the same wavelengths. Calculation of the total anthocyanins content of LPE follows the equation of:

Anthocyanin content (mg/g of LPE) = A × MW × 100/ε(1)

where A denotes absorbance = (A_515_ − A_700_)_pH 1.0_ − (A_515_ − A_700_)_pH 4.5_; MW is the molecular weight specific to cyanidin-3-glucoside (449.2); and ε stands for the molar absorptivity of cyanidin-3-glucoside (26,900). The total anthocyanin content was in the form of mg C-3-G E/g LPE. We repeated the experiment three times independently.

### 2.7. Total Proanthocyanidin Determination

The proanthocyanidins content of LPE was estimated using the previously method [2]. In brief, the concentrations of 1 mg/mL epicatechin stock solution and epicatechin working solution standard concentrations of 0–100 μg/mL were prepared in 50% (*v/v*) aqueous methanol solution. The results are expressed as milligrams of epicatechin equivalents per gram of LPE (mg EE/g LPE). We mixed 0.5 m of epicatechin standard solution with 2.5 mL of aqueous vanillin (30 mg/mL). Shaking and 2 min of rest were followed by the addition of 2.5 mL of H_2_SO_4_–methanol solution (30% *v/v*). After the solution was stirred, the mixture received 20 min of incubation at 30 °C in a dark place. Subsequently, the absorbance was examined at 500 nm. The LPE working solution (concentration: 0.1 mg/mL) was prepared in 50% ethanol, and the above method was also adopted to examine the total proanthocyanidin content. This experiment was repeated three times independently. The EE standard curve and regression equation were established and the total proanthocyanidin content of LPE was calculated according to the EE standard curve.

### 2.8. Quantification of Individual Compound

Existing research of litchi pericarp revealed the presence of polyphenols, mainly including (+)-catechin, rutin, (-)-epigallocatechin, 3,4-dihydroxybenzoic acid, quercetin, epicatechin, epigallocatechin gallate, procyanidin A2, procyanidin B2, procyanidin A1, and procyanidin B1. A liquid chromatography-tandem mass spectrometry (LC-MS/MS) method for quantifying compounds in LPE was proposed [16]. We dissolved the LPE and reference materials in an aqueous solution of 50% methanol. The analysis was conducted on an Agilent Eclipse Plus C18 (50 mm × 2.1 mm, 1.8 μm), and the flow rate was obtained as 400 μL/min with a methanol–0.1% aqueous formic acid solution as the mobile phase in gradient elution mode. The injection volume reached 1.0 μL, and the column temperature was 35 °C. A triple quadrupole tandem mass spectrometer was used for the detection (Agilent 6430, Agilent Technologies Co. Ltd.,SC, USA) with an electrospray ionization (ESI) source using multiple reaction monitoring (MRM) mode. The positive and negative ion modes were used to determine different compounds with an MRM mode. The capillary voltage reached 3500 V under positive ion mode and reached 3000 V under negative ion mode.

### 2.9. Determination of DPPH Radical Scavenging Activities

A DPPH free radical scavenging capacity test was performed using a method proposed before with made some modifications [17]. The process is presented as follows. The 100 μg/mL LPE stock solution and the 0–1.0 μg/mL LPE working solution were prepared in 50% (*v*/*v*) aqueous ethanol solution. Subsequently, 500 µL of LPE solution (0, 0.2, 0.4, 0.6, 0.8 and 1.0 µg/mL) was mixed with 500 µL of DPPH reagent (0.1 mM, dissolved in absolute ethanol). After 30 min of incubation in the dark at 37 °C, the absorbance was examined at 517 nm. Ascorbic acid and luteolin were treated as the positive controls. We carried out experiments in triplicates. The antioxidant capacity was obtained as follows:

DPPH radical scavenging activity (%) = (Ac − As)/Ac × 100% (2)

where “Ac” denotes the absorbance of the blank control and “As” denotes that of the sample solution.

### 2.10. ABTS Radical Scavenging Activities

The ABTS method was conducted in accordance with the previous methods [18]. Stock solutions of ABTS (7 mM, dissolved in an aqueous solution) and K_2_S_2_O_8_ (140 mM, in an aqueous solution) were prepared. The K_2_S_2_O_8_ solution (176 µL) was introduced into the ABTS solution (10 mL). The mixture solution was stored in the dark at 27 °C for 12 h, and double distilled water was used for diluting to achieve an absorbance of 0.70–0.75 at 734 nm. The 100 μg/mL LPE stock solution and the 0–1.0 μg/mL LPE working solution were prepared in 50% (*v/v*) aqueous ethanol solution. The fundamental steps are presented as follows. Briefly, 0.2 mL of LPE solution (0, 0.2, 0.4, 0.6, 0.8, and 1.0 µg/mL) was mixed with 2 mL ABTS mixture solution. After the solution was stirred, the mixture underwent 30 min of incubation at 27 °C without exposure to light. Next, the absorbance was examined at 734 nm. Ascorbic acid and luteolin served as the positive controls. The experiments were performed in triplicates. The antioxidant capacity is expressed as follows:

ABTS radical scavenging activity (%) = (Ac − As)/Ac × 100% (3)

Thereinto, “Ac” denotes the absorbance of the blank control and “As” represents that of the sample solution.

### 2.11. In Vivo Antioxidant Tests

The antioxidant activity of LPE on the D-galactose-induced aging model mice was evaluated [19].

#### 2.11.1. Animals

All animal procedures gained approval from the Research Ethics Committee of the Guangxi Institute of Botany, Guangxi Zhuang Autonomous Region and the Chinese Academy of Science (GXZW2020051001). One hundred four-week-old male C57BL/6J mice were obtained from Hunan SJA Laboratory Animal Co., Ltd., Changsha. The mice were kept in an environment at 22 ± 2 °C and 45–55% relative humidity in a 12 h dark/12 h light cycle. The mice were fed a standard commercial chow diet (Hunan SJA Laboratory Animal Co., Ltd., Changsha, China). We acclimated mice for seven days prior to the experiments.

#### 2.11.2. Experimental Design

After the acclimation period of 7 days, 400 mg/Kg∙d D-galactose was subcutaneously injected into the mice, except for the control group (CG), for 8 weeks to build an oxidative stress animal model. The CG was injected with equal volume of normal saline as the vehicle control. After 8 weeks, the blood samples were collected from the retro-orbital sinus of all mice. The serum MDA levels were obtained using the commercial kits in accordance with the instructions. As found, the model mice presented obviously higher serum MDA levels relative to the CG mice, such that the oxidative stress model was well built. Subsequently, 60 model mice with the highest serum MDA levels were selected for the next step of the experiment. After 3 days of acclimatization, 60 model mice were randomly assigned to five groups in accordance with their serum MDA levels, each of which contained 12 mice. To be specific, the model group (MG) was treated with normal saline, the positive group (PG) was given 50 mg/Kg∙d VE, the low-dose LPE group (L-LPE) was treated with 100 mg/Kg∙d LPE, the mid-dose LPE group (M-LPE) was given 200 mg/Kg∙d LPE, and the high-dose LPE (H-LPE) group was given 400 mg/Kg∙d LPE. 400 mg/Kg∙d D-galactose was subcutaneously injected into the mice for 30 consecutive days, and equal volume of normal saline was adopted for treating the controls. The mice in the CG and MG were treated by using normal saline, and the mice in the other groups received 30 consecutive days of intragastric gavage using VE or LPE. We weighted the animals once weekly and adjusted the treatment dosage in accordance with mice body weight.

#### 2.11.3. Observation of Animals

During the experiment period, the mental condition, behavioral activity, and appearance of the animals in each group were observed each week.

#### 2.11.4. Blood and Tissue Sampling

Thirty days later, pentobarbitol sodium (intraperitoneally, 50 mg/kg) was adopted for anesthetizing the mice. The collected blood samples underwent 15 min of centrifugation at 4 °C after the loss of consciousness of the mice. The serum was stored at −20 °C to be used for measuring the antioxidant capacity biochemical parameters. Cervical dislocation was adopted for sacrificing animals, and then we stripped the liver and kidney. After the weighting of respective whole organ, we obtained the organ index as follows: organ index = organ mass (mg)/mice body weight (g). Parts of the same region of each organ were homogenized using a glass homogenizer, followed by 10 min of centrifugation at 3500 rpm at 4 °C. We then collected the supernatants for analyzing antioxidant capacity indexes.

#### 2.11.5. Analysis of Serum and Tissue Antioxidant Capacity Indexes

Serum and tissue antioxidant capacity indexes SOD, MDA, GSH, and protein carbonyl were obtained using the commercial kits following the method described by the manufacturer.

### 2.12. Statistical Analysis

All experimental data are expressed as means ± standard deviation (SD) or means ± standard errors (SE). The mean values of multiple samples were compared using One-way analysis of variance (ANOVA). GraphPad Prism 8 (GraphPad Software, San Diego, CA, USA) program software was employed for the statistical analysis, and *p* < 0.05 reported statistical significance.

## 3. Results

### 3.1. Determination of Total Polyphenol, Total Flavonoid, Total Anthocyanin and Total Proanthocyanidin Contents

As depicted in Table 1, the extraction rate of the fresh litchi pericarp was 4.76%. The LPE contains 75.06% of total polyphenol, 13.98% of total flavonoid, 5.51% of total anthocyanin and 28.53% of total proanthocyanidin. The polyphenol compounds in LPE were analyzed using the liquid chromatography-tandem mass spectrometry (LC-MS/MS) method. As shown in Table 1, the LPE contains 2.345% of procyanidin A2, 0.925% of procyanidin B2, 0.363% of epicatechin, 0.296% of rutin, and 0.256% of epicatechin. The levels of quercetin, (-)-epigallocatechin, procyanidin A1, epigallocatechin gallate, 3,4-dihydroxybenzoic acid, and procyanidin B1 in the LPE were very low.

### 3.2. Radical Scavenging Activity of LPE

**Figure 1:**
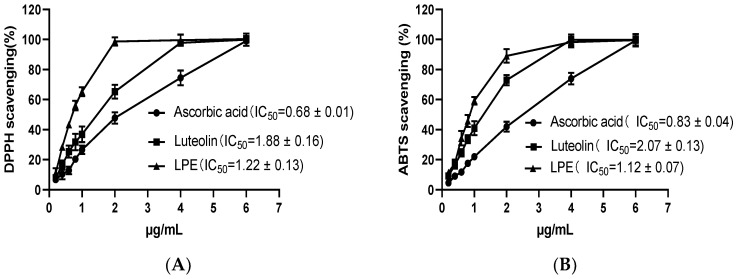
DPPH and ABTS assays of radicals scavenging activity of LPE: (**A**) DPPH radical scavenging activities; (**B**) ABTS radical scavenging activities. Values are expressed as the mean of triplicate experiments ± standard error.

Figure 1 gives the DPPH and ABTS radical scavenging activity test results, thus demonstrating the antioxidant effect of LPE and the positive control (ascorbic acid and luteolin). Different doses of LPE, ascorbic acid, and luteolin are capable of scavenging the DPPH and ABTS radicals. The scavenging effects will increase as the sample concentrations elevates. The general rule for LPE to clear DPPH and ABTS free radicals was that the clearance rate tended to increase with the increase in the LPE concentration (0.2–1.0 µg/mL), and when the concentration reached 2.0 µg/mL, the clearance rate leveled off. The general rule for ascorbic acid to clear DPPH and ABTS free radicals was that the clearance rate increased gradually with an increase in ascorbic acid concentration (0.2–6.0 µg/mL). In luteolin, the DPPH and ABTS radical scavenging activity peaked at 4.0 µg/mL, and stabilized thereafter. The IC_50_ value for the scavenging activity of DPPH free radical in LPE, lower than ascorbic acid and luteolin. Furthermore, the IC_50_ value specific to the scavenging activity of the ABTS free radical was lower in LPE than in ascorbic acid and luteolin.

### 3.3. The Effects of LPE on Body Weight, Organ Coefficient and Daily Behavior

**Figure 2:**
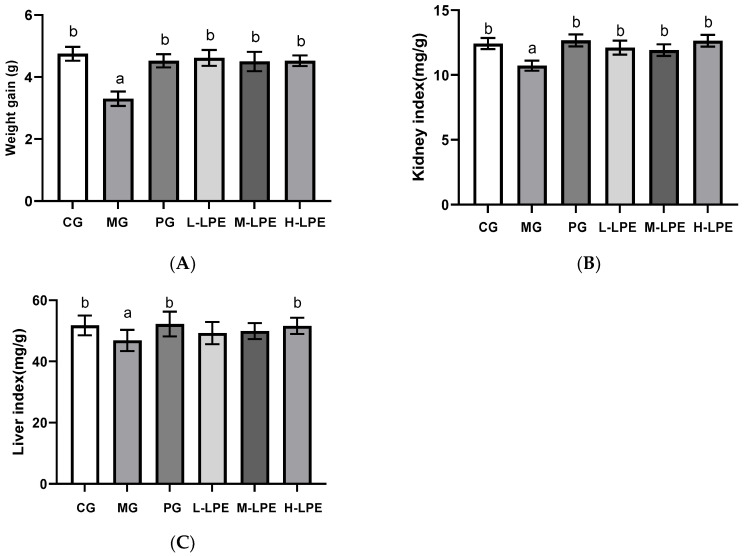
Effects of LPE on body weight, organ indexes in aging mice induced by D-galactose: (**A**) effects of LPE on body weight; (**B**) effects of LPE on kidney index; (**C**) effects of LPE on liver index. Values are expressed as mean ± standard error (*n* = 12 in each group). Values with different superscripts showed a difference with statistical significance, *p* < 0.05, “a” as a statistical difference compared with the blank control group, “b” as a statistical difference compared with the model group.

**Figure 3:**
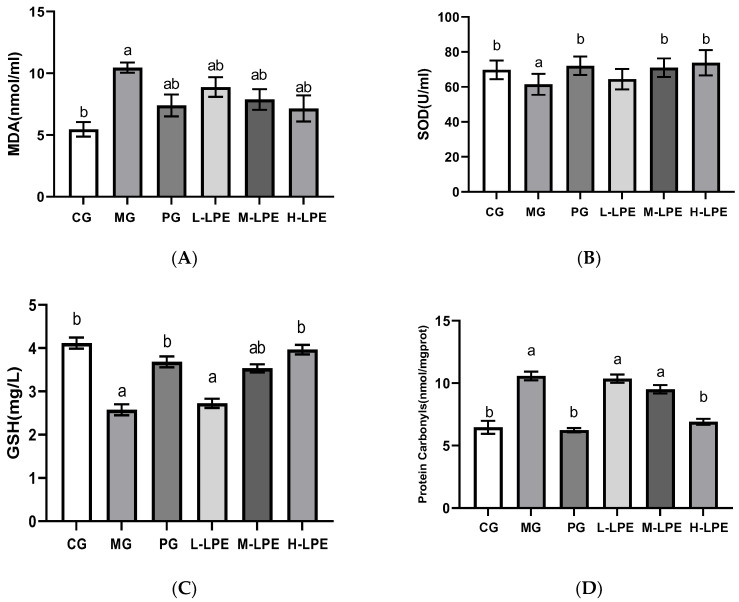
Levels of MDA, SOD, GSH and protein carbonyls in serum of mice: (**A**) MDA content in serum of mice; (**B**) SOD activity in serum of mice; (**C**) GSH content in serum of mice; (**D**) protein carbonyls content in serum of mice. Values are expressed as mean ± standard error (*n* = 12 in each group). Values with different superscripts showed a difference with statistical significance, *p* < 0.05, “a” as a statistical difference compared with the blank control group, “b” as a statistical difference compared with the model group.

During the 30-day period of the experiment, the behavioral activities and daily appearance of the animals were observed. The results showed that for all mice there were no daily behavioral changes, including anti-feeding and vomiting. The CG mice maintained a good mind stage, meanwhile being active, lively, and agile. The MG mice showed symptoms of aging, such as response lag, slow movement, drooping spirit, and slowly reduced food intake. Relative to the MG mice, mice in treatment group presented receded senescence characteristics. Body weight changes are capable of reflecting animal development and growth, and the adverse effects of drugs on the body [20]. The body weight data are shown in Figure 2. Relative to the CG, the mice in the MG showed weight loss after D-galactose injection (*p* < 0.05). The aging mice treated with various doses of LPE or VE for 30 days underwent a reversal of the weight losses that D-galactose had induced (*p* < 0.05). Organ indexes are capable of directly indicating the size changes of the experimental animal organ, as well as physical health [21]. According to the experimental results (Figure 3), the aging mice of the MG presented obviously lower kidney and liver coefficients relative to the CG (*p* < 0.05), which indicated that the mice receiving D-galactose injection underwent organ aging. In addition, the treatment group exhibited remarkably higher kidney index relative to the model group (*p* < 0.05). Moreover, compared with the MG mice, the mice treated with L-LPE or VE for 30 days underwent a reversal of the liver losses that D-galactose had induced (*p*< 0.05), whereas this trend in the L-LPE and the M-LPE mice did not achieve statistical significance.

### 3.4. Levels of MDA, SOD, GSH and Protein Carbonyls in Serum of Mice

As depicted in Figure 3A,D, the MDA and protein carbonyls levels in the serum presented an obvious elevation in the MG mice relative to the CG mice (*p* < 0.05). In contrast, all LPE groups decreased the serum MDA levels in mice receiving D-galactose treatment (*p* < 0.05), showing an obvious dose–effect relationship. As depicted in Figure 3D, relative to the MG, the mice in the H-LPE presented a loss in the serum protein carbonyls level (*p* < 0.05). However, this trend in L-LPE and M-LPE did not achieve statistical significance. The serum MDA and protein carbonyls levels in the PG exhibited an obvious decrease relative to the MG (*p* < 0.05). As illustrated in Figure 3B,C, the serum SOD and GSH levels were significantly decreased in the MG mice compared with the CG mice (*p* < 0.05). Relative to the MG, the SOD activity and GSH content in the serum of the mice treated with the LPE intervention groups (200 and 400 mg/Kg∙d) presented a statistically significant increase (*p* < 0.05). The PG presented remarkably increased serum SOD and GSH levels, relative to the MG (*p* < 0.05).

### 3.5. Levels of MDA, SOD, GSH and Protein Carbonyls in Liver of Mice

**Figure 4:**
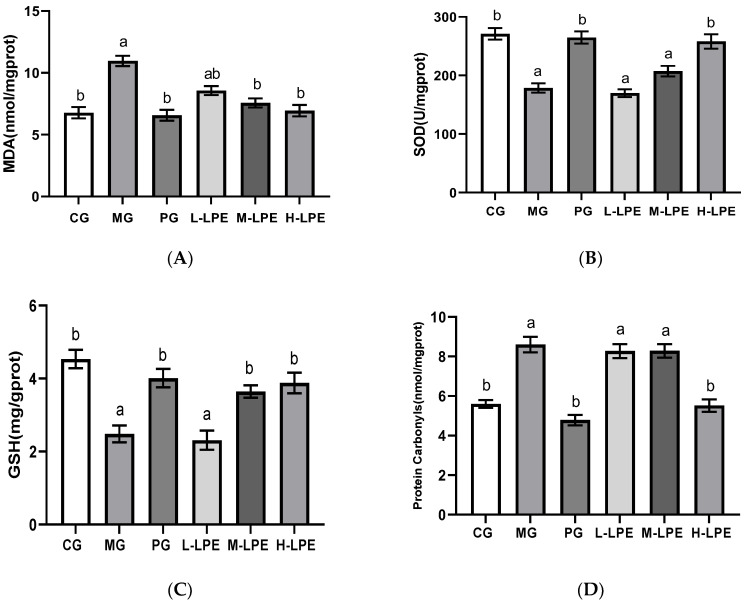
MDA, SOD, GSH, and protein carbonyls levels in the liver of mice: (**A**) MDA content in the liver of mice; (**B**) SOD activity in the liver of mice; (**C**) GSH contents in the liver of mice; (**D**) protein carbonyls content in the liver of mice. Values are expressed as mean ± standard error (*n* = 12 in each group). Values with different superscripts showed a difference with statistical significance, *p* < 0.05, “a” as a statistical difference compared with the blank control group, “b” as a statistical difference compared with the model group.

As observed visually in Figure 4, the MG mice after subcutaneous injection of 400 mg/Kg∙d D-galactose presented remarkably decreased antioxidant capacity of liver, relative to the CG mice (*p* < 0.05). As depicted in Figure 4A, after LPE treatment (100, 200, and 400 mg/Kg∙d) or VE, the content of MDA in aging mice livers exhibited a gradual decrease (*p* < 0.05). As can be seen in Figure 4B, the administration of high-dose litchi pericarp extracts (LPE) or VE led to increase in SOD activity in the liver (*p* < 0.05), and the low dose and medium dose of LPE had no effect on liver SOD activity. As depicted in Figure 4C, the GSH content in the liver of the PG, the M-LPE, and the H-LPE presented an obvious increase, relative to the MG (*p* < 0.05), whereas the GSH of the L-LPE was not affected. As depicted in Figure 4D, after the administration of high-dose LPE or VE, the content of protein carbonyls in aging mice livers decreased (*p* < 0.05), and the low and medium doses of LPE had no effect on protein carbonyls.

### 3.6. Levels of MDA, SOD, GSH and Protein Carbonyls in Kidney of Mice

**Figure 5:**
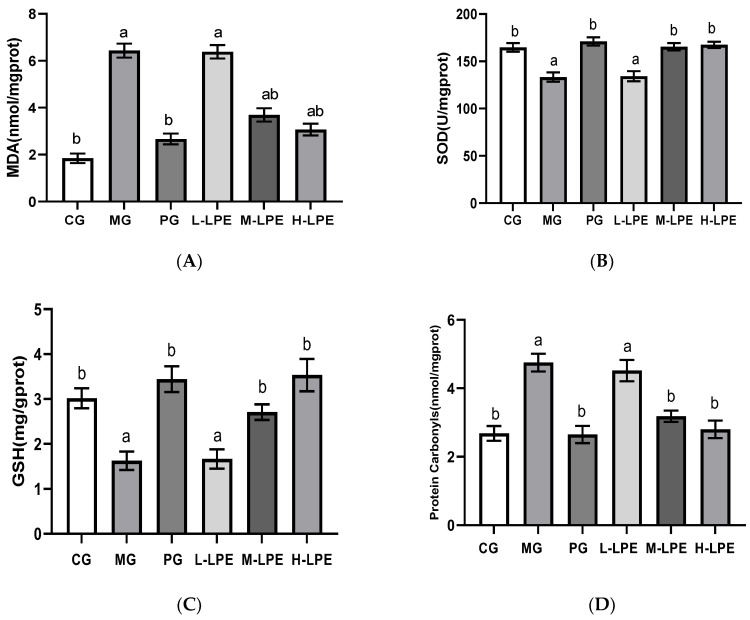
MDA, SOD, GSH, and protein carbonyls levels in the kidney of mice: (**A**) MDA contents in the kidney of mice; (**B**) SOD activity in the kidney of mice; (**C**) GSH contents in the kidney of mice; (**D**) protein carbonyls content in the kidney of mice. Values are expressed as mean ± standard error (*n* = 12 in each group). Values with different superscripts showed a difference with statistical significance, *p* < 0.05, “a” as a statistical difference compared with the blank control group, “b” as a statistical difference compared with the model group.

Figure 5 shows that the kidney of mice from the MG reported an obviously weakened antioxidant capacity relative to the CG (*p* < 0.05). As depicted in Figure 5A,D, after the administration of mid–dose LPE, high–dose LPE, and VE, the contents of MDA and protein carbonyls in the kidney were decreased, relative to the MG (*p* < 0.05), but the mice treated with low-dose LPE demonstrated no statistically significant change in kidney MDA or protein carbonyls concentrations. As displayed in Figure 5B,C, the SOD and GSH levels exhibited a remarkable decline in the MG mice relative to the CG mice (*p* < 0.05). In contrast, mid-dose LPE, high–dose LPE and vitamin E administrations increased the SOD and GSH levels in mice receiving D-galactose treatment (*p* < 0.05), while low-dose LPE had no effect on the above two indices.

## 4. Discussion

The Litchi pericarp contain numerous health-enhancing components with great potential application in the food industries. Therefore, the utilization of valuable ingredients from litchi processing by-products in food additives is a very important area for researchers. The quantification of these active ingredients in relation to the physiological functions of Litchi pericarp needs to further be studied. Additionally, the development of efficient, safe, and convenient extraction methods should be considered.

Polyphenol content in extracts is related to their antioxidant function. The recovery of the polyphenol compounds from natural plant sources could increase their commercial value. Thus, it is very important to optimize the extraction process conditions to obtain a high extraction rate for polyphenols. In general, drying of the sample was required till extraction, but drying remarkably impacted the bioactive activity and the phytochemical compositions. An existing study showed how different drying treatments affected polyphenols in the pericarp of three longan cultivars, finding that the content of polyphenols followed an increasing order for the drying methods as follows: solar radiation < microwave radiation < lyophilization [22]. The previous study further showed that there was no difference in the content of polyphenols between the lyophilized pericarp and fresh pericarp [22]. This study used lyophilized fresh litchi pericarp for extraction. In this study, the extraction rate for LPE was approximately 4.76% in fresh litchi pericarp, and the content of total polyphenol was approximately 750.6 mg GAE/g LPE. Previous work revealed that litchi pericarp was extracted with 70% ethanol, and the content of total polyphenol was approximately 357.9 mg GAE/g extracts [12]. In addition, the litchi pericarp of nine litchi varieties was studied for extraction with acetone. The total polyphenol content ranged from 9.39 to 30.16 mg GAE/g fresh litchi pericarp, and the average content was 16.27 mg GAE/g fresh litchi pericarp being reported [2]. In this study, the content was approximately 35.7 mg GAE/g fresh litchi pericarp. Current litchi pericarp polyphenol compounds extractions use ethanol, methanol, or acetone as a solvent for extraction. The use of organic solvent extraction often results in higher costs. In addition, an organic solvent may produce harmful effects when its residue remains in the extracts. In this study, the litchi pericarp was extracted with a saturated aqueous Na_2_CO_3_ solution, such that this extraction method was more efficient, convenient, and safer than the former organic solvent methods. Proanthocyanidins are also known as condensed tannins [23]. Proanthocyanidins possess many biological activities, such as antioxidation, anti-inflammation, antiangiogenesis, antitumor, and antiproliferation activities. In this study, the content of total proanthocyanidin was approximately 285.3 mg EE/g LPE. Using such an approach, LPE with a high proanthocyanidins content can be obtained. In this work, we determined the contents of the main monomer in LPE, as depicted in experimental results: procyanidin A2, procyanidin B2, epicatechin, rutin, and catechin are the major polyphenol compounds of litchi pericarp, consistent with the results of previous studies [2,24].

DPPH, as a steady free radical, has an unpaired electron. When there is a free radical scavenger, DPPH will accept an electron or a hydrogen atom, which can be employed to evaluate antioxidant function [25]. ABTS free radical cation is blue in color. When there are antioxidants, free radical scavengers in the sample reduce the blue color ABTS free radicals to a colorless reduced ABTS form. The ABTS test is a convenient method for examining antioxidant capacity [26]. The results of the DPPH and ABTS tests suggest that LPE has high antioxidant activity. Ascorbic acid and luteolin are vital antioxidants, whereas LPE exhibits excellent DPPH and ABTS radical scavenging activity higher than that of standard ascorbic acid and luteolin. Existing research has suggested that the litchi pericarp polyphenol extracts exhibit a high antioxidant capacity, the IC_50_ values for DPPH and ABTS reach 2.29 μg/mL and 7.14 μg/mL in extracts, respectively, and the content of total polyphenol is approximately 357.9 mg GAE/g extracts [12]. As mentioned above, in this study, the IC_50_ values for the DPPH and ABTS were 0.68 μg/mL and 0.83 μg/mL in LPE, respectively, and the content of total polyphenol was approximately 750.6 mg GAE/g LPE. In the DPPH and ABTS tests, the activity of LPE was approximately 2.37-fold and 7.60-fold higher than in previous extracts. Besides, the content of total polyphenol in LPE was approximately 1.10-fold higher than in previous extracts. The result of this data analysis suggests that the increase in total polyphenols content can increase the antioxidant capacity significantly. This study confirms that LPE exhibits excellent antioxidant function in vitro.

Although LPE showed excellent antioxidant activity in vitro, the antioxidant activity will not necessarily be the same in vivo as in vitro. The antioxidation of compounds is affected by many factors; many compounds that appear to have good activity in vitro show no activity in vivo. Accordingly, this study still needs to focus on the evaluation of LPE antioxidant function in vivo. In this study, an oxidative aging mice model induced by D-galactose assisted in evaluating LPE antioxidant function in vivo. D-galactose is a compound capable of inducing aging, and aging induced in a mice model is similar to natural aging, such that aging model mice are widely used to study aging [27]. This aging model was first reported in 1985, when scientists revealed obvious aging signs in mice after eight weeks of treatment using D-galactose [28]. This is due to the reduction of intracellular galactose to galactitol, which is incapable of being further degraded. Hence, the galactitol accumulated in cells results in metabolic disorders, produces reactive oxygen species (ROS), and causes causing oxidative stress, thereby inducing oxidative aging [29]. Thus, the D-galactose-induced aging model is also an oxidative stress model.

ROS are byproducts of enzymatic reactions in cells. ROS initiate oxidation in the body, causing metabolic disorder and numerous diseases [30]. Antioxidants are classified into two types, including exogenous and endogenous. Endogenous antioxidants comprise a wide variety of enzymes (e.g., catalase, glutathione peroxidase and SOD) [31]. SOD is a very important endogenous antioxidant since it is the only enzyme defense system that decomposes superoxide anion into H_2_O_2_ [32,33]. Thus, SOD can eliminate ROS and maintain the physiological redox balance, consequently protecting the body from harmful oxidative stress [34]. It has long been realized that a high ROS level can lead to damaged lipids. Lipid peroxidation products commonly exert cytotoxic effects. On that basis, the control of the production of lipid peroxidation products remarkably helps to prevent diseases. MDA is one of the final products of lipid peroxidation [35]. Accordingly, MDA levels are capable of directly indicating the levels of lipid peroxidation, and thus the degree of body damage is indirectly indicated. ROS can cause several types of damages, and one of the above is protein oxidation. Protein carbonyls have been confirmed as a major product of protein oxidation and an irreversible protein modification form. Protein carbonyls can be formed in the early stages of protein oxidation and are not a result of one specific oxidation, such that they have been extensively employed to indicate protein oxidation [36]. Glutathione consists of glycine, cysteine, and glutamate and it is an important antioxidant in cells [37]. Glutathione can greatly help to maintain the intracellular detoxification of electrophilic metabolites and thiol status. Glutathione usually exists in two different forms, including oxidized glutathione and GSH states, whereas the majority of it has been detected in a reduced form in the body. GSH plays an antioxidant function by scavenging oxygen free radicals during lipid peroxide. MDA, SOD, GSH, and protein carbonyls were chosen as the important indexes to evaluate antioxidant function. The above indicators were also recommended for use in studying oxidation by the China Food and Drug Administration. D-galactose causes oxidation/anti-oxidation system imbalance in the body [38,39]. Consistent with existing research [19], this study found that the mice injected with D-galactose had reduced the activity of antioxidant enzymes. For instance, decreased SOD activity, exacerbated lipid peroxidation, manifested as an increase in the levels of MDA, and induced protein oxidation, manifested as the accumulation of protein carbonyls. Furthermore, the scavenging of oxygen free radicals was also reduced, with the decrease of the GSH content. Accordingly, the aging model was successfully built. In this study, LPE significantly increased the SOD activity and GSH contents in the liver, kidney, and serum of the aging mice, and reduced the contents of MDA and protein carbonyls in the liver, kidney, and serum, thus suggesting that LPE led to the increased activity of antioxidant enzymes, the reduced protein oxidation and lipid peroxidation, and a greater capacity to scavenge free radicals. The data analysis results show that procyanidin A2, procyanidin B2, epicatechin, rutin, and catechin are the major polyphenol compounds of LPE. It was reported that these compounds had an antioxidation effect [40,41,42,43,44]. Procyanidin B2 significantly decreased the content of MDA and enhanced the activities of antioxidant enzymes SOD in carbon tetrachloride-induced acute liver injury mice [41]. Epicatechin enhanced antioxidant activities through intestinal microbiota [42]. Rutin exhibits significant scavenging properties on oxygen radicals both in vitro and in vivo [43]. Catechin may reduce hepatic fibrosis by suppressing oxidative stress [44]. In conclusion, there is a relationship between the antioxidant effects in oxidative stress model mice and these compounds. Moreover, VE has been shown to have good antioxidant effects in vivo, and a considerable number of antioxidant studies have selected VE as a positive drug [38,39]. Consistent with existing research, this study found that VE can significantly improve the antioxidant capacity of aging mice. The above results suggest that LPE has a good antioxidant effect on the body.

## 5. Conclusions

In this study, a novel extraction method of LPE was proposed, and the high-purity litchi pericarp polyphenols were obtained. In quantitative analyses of the major monomers in the LPE, procyanidin A2, procyanidin B2, epicatechin, rutin, and catechin were found to be major polyphenol compounds of LPE. The results of the ABTS and DPPH tests showed that the LPE displays excellent free radical scavenging activity, indicating the good antioxidant capacity of LPE in vitro. The results of this study indicated weakened SOD activity and reduced GSH content, alongside increased MDA and GSH contents in the mice injected with D-galactose. In the aging mice, the above symptoms were improved after the LPE treatment was performed. LPE significantly increased the SOD activity and the GSH content, and reduced MDA and protein carbonyl contents. Thus, LPE can be a promising source of dietary antioxidants.

## Figures and Tables

**Table 1:** The extraction rate of the fresh litchi pericarp and determination of polyphenols, flavonoids, anthocyanins and proanthocyanidins contents and quantitation of 11 individual compounds in LPE.

Item	Litchi Pericarp
Extraction rate (mg LPE/g fresh litchi pericarp)	47.6 ± 4.3
Total polyphenol (mg GAE/g LPE)	750.6 ± 22.4
Total flavonoid (mg RE/g LPE)	139.8 ± 3.5
Total anthocyanin (mg C-3-GE/g LPE)	55.1 ± 0.7
Total proanthocyanidin (mg EE/g LPE)	285.3 ± 9.8
Procyanidin A2 (mg/g LPE)	23.45 ± 0.82
Procyanidin B2 (mg/g LPE)	9.25 ± 0.16
Epicatechin (mg/g LPE)	3.63 ± 0.17
Rutin (mg/g LPE)	2.96 ± 0.13
(+)-Catechin (mg/g LPE)	2.56 ± 0.10
Quercetin (mg/g LPE)	0.53 ± 0.08
(-)-Epigallocatechin (mg/g LPE)	0.40 ± 0.03
Procyanidin A1 (mg/g LPE)	0.25 ± 0.03
Epigallocatechin gallate (mg/g LPE)	0.18 ± 0.03
3,4-Dihydroxybenzoic acid (mg/g LPE)	0.08 ± 0.01
Procyanidin B1 (mg/g LPE)	0.0014 ± 0.0001

Values are expressed as the mean of triplicate experiments ± standard deviation. Total polyphenol was obtained as gallic acid equivalent (mg GAE/g LPE). Total flavonoid was obtained as rutin equivalent (mg RE/g LPE). Total anthocyanin was obtained as cyanidin-3-glucoside equivalents (mg C-3-G E)/g LPE. Total proanthocyanidin was obtained as epicatechin equivalent (mg EE/g LPE).

## Data Availability

Data is contained within the article.
